# Supplementary material for: Hidden prevalence of deletion-inversion bi-alleles in CRISPR-mediated deletions of tandemly arrayed genes in plants
Source: Nat Commun. 2023 Oct 25;14:6787. doi: 10.1038/s41467-023-42490-1 (PMC10600118; doi:10.1038/s41467-023-42490-1)
Supplement: Supplementary file 1 — Supplementary Information [file 41467_2023_42490_MOESM1_ESM.pdf]

Hidden prevalence of deletion-inversion bi-alleles in CRISPR-mediated deletions of tandemly arrayed genes in plants

Liu *et al.*

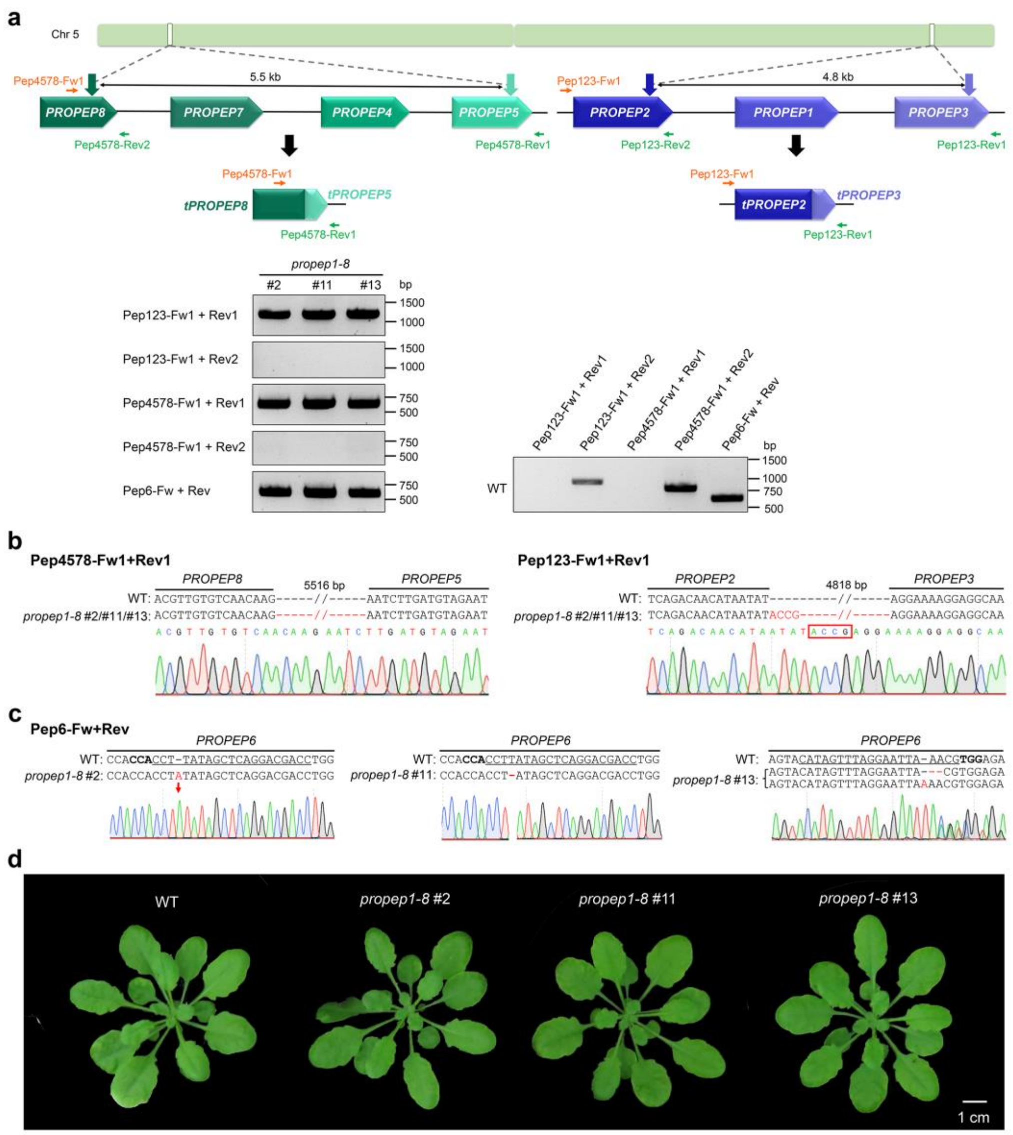

Supplementary Fig. 1. Two-tier PCR-based genotyping of *atpropep1-8* mutant lines.

a, Standard two-tier PCR-based genotyping identifies three homozygous *atpropep1-8* mutant lines (#2, #11, #13) in the T_1 generation. The upper panel shows the diagram of the *AtPROPEP8/7/4/5* and *AtPROPEP8/7/4/5* genomic loci with the information of gRNAs and genotyping primers. Fw, forward primer. Rev, reverse primer. Experiments were repeated twice with similar results. **b**, Sanger sequencing results validate the deletions of *AtPROPEP8/7/4/5* and *AtPROPEP2/1/3* loci in all three *atpropep1-8* mutant lines. Deletions are indicated by red dashes, while insertions are colored in red or indicated by a red box. **c**, Sanger sequencing results validate different loss-of-function mutations of *AtPROPEP6* in three *atpropep1-8* mutant lines. The sequences of the antisense strand containing the target of gRNA-Pep6.2 are shown for the lines #2 and #11, whereas the sequence of the sense strand containing the target of gRNA-Pep6.1 is shown for the line #13. Bold letters mark PAMs and target sequences are underlined. **d**, All three *atpropep1-8* T_1 lines are phenotypically indistinguishable from WT plants at 4 weeks old. Source data are provided as a Source Data file.

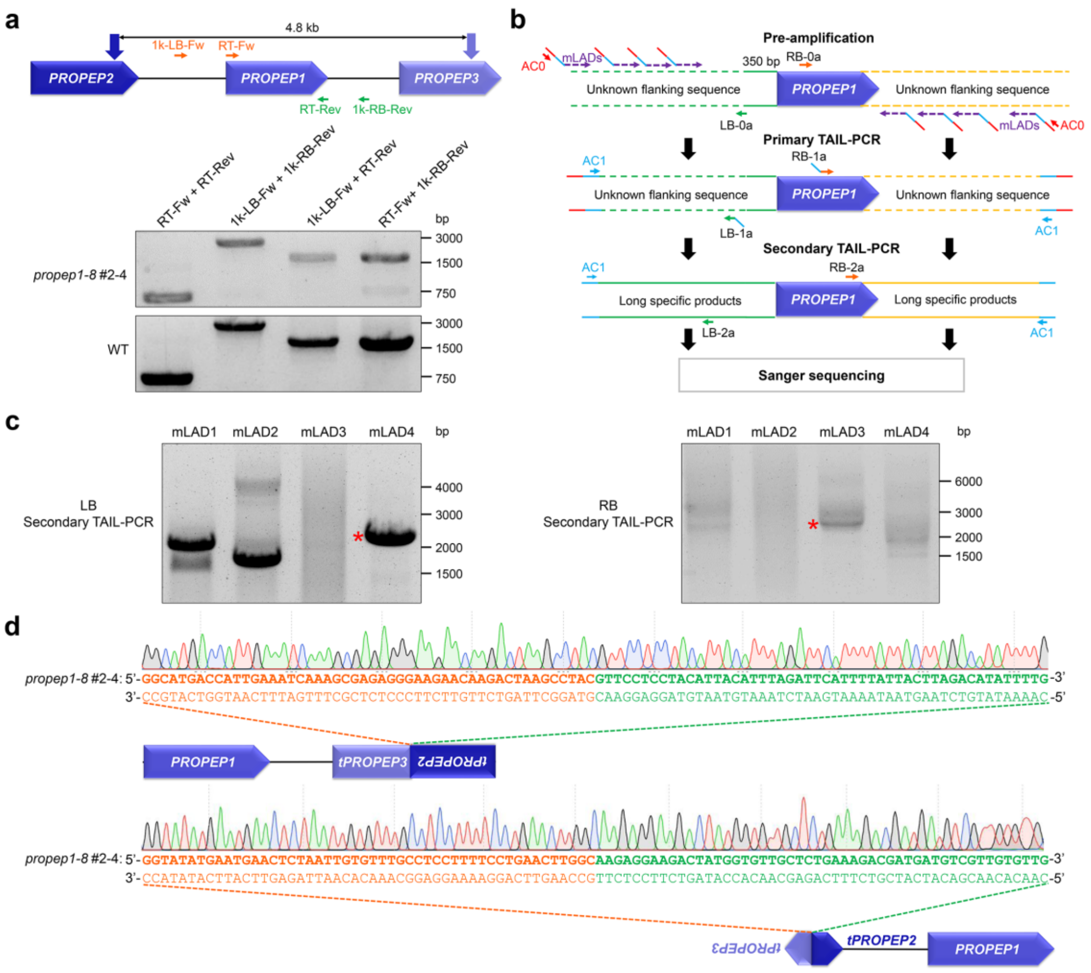

Supplementary Fig. 2. Identification of the genomic re-insertion site of the deleted *AtPROPEP2/1/3* fragment.

a, PCR indicates the re-insertion of the deleted *AtPROPEP2/1/3* fragment in the genome in *atpropep1-8* #2-4 plants. PCR primers used are shown in the diagram. Fw, forward primer. Rev, reverse primer. The primers RT-Fw and RT-Rev target the coding region of *AtPROPEP1*, while the primers 1k-LB-Fw and 1k-RB-Rev target the 1-kb flanking region of *AtPROPEP1*. Experiments were repeated twice with similar results. **b**, Schematic diagram of TAIL-PCR used for identifying the genomic re-insertion site of the deleted *AtPROPEP2/1/3* fragment. **c**, Agarose gel electrophoresis for LB and RB secondary TAIL-PCR amplicons. The products marked with red asterisk were subjected to Sanger sequencing. **d**, Representative Sanger sequencing results reveal the inverted re-insertion of the deleted *AtPROPEP2/1/3* fragment between the gRNA-Pep2 and gRNA-Pep3 target sites in *atpropep1-8* #2-4 plants. The sequences of *AtPROPEP3* are in orange, while the sequences of *AtPROPEP2* are in green. Source data are provided as a Source Data file.

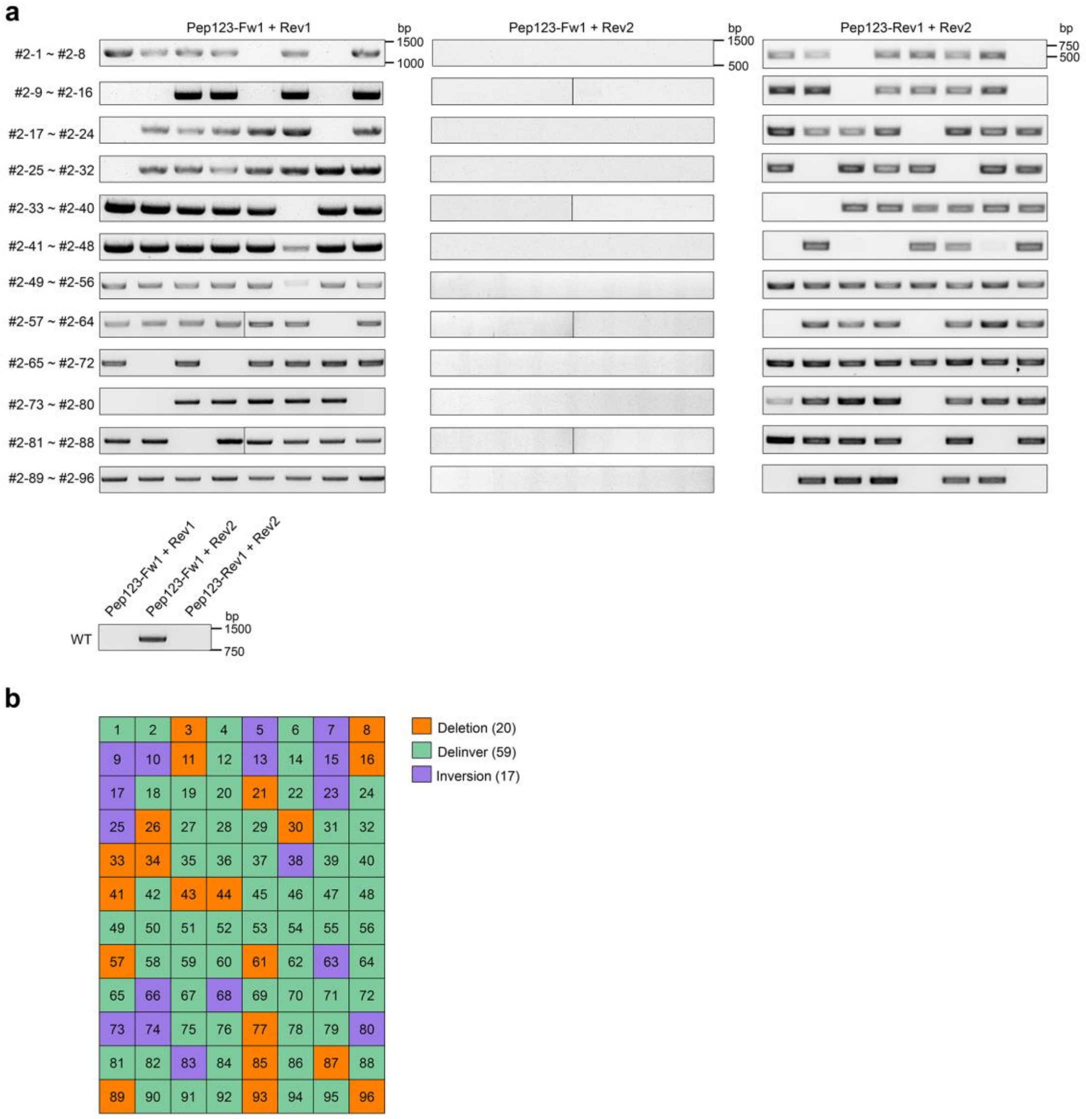

Supplementary Fig. 3. Genetic heterogeneity at the *AtPROPEP2/1/3* locus in T_2 progeny of the *atpropep1-8* mutant line #2.

a, Three-tier PCR-based genotyping of T_2 progeny from the *atpropep1-8* mutant line #2. The primers Pep123-Fw1/Rev1 were used in the tier-1 PCR (left panel) to detect deletions of *AtPROPEP2/1/3*. The primers Pep123-Fw1/Rev2 were used in the tier-2 PCR (middle panel) to detect *AtPROPEP2/1/3* without deletions. The primers Pep123-Rev1/Rev2 were used to in the tier-3 PCR (right panel) to detect inversions of *AtPROPEP2/1/3*. PCR amplicons from WT plants served as controls. Experiments were repeated twice with similar results. **b**, Summary of the genotypes of individual progeny tested in **a**. Source data are provided as a Source Data file.

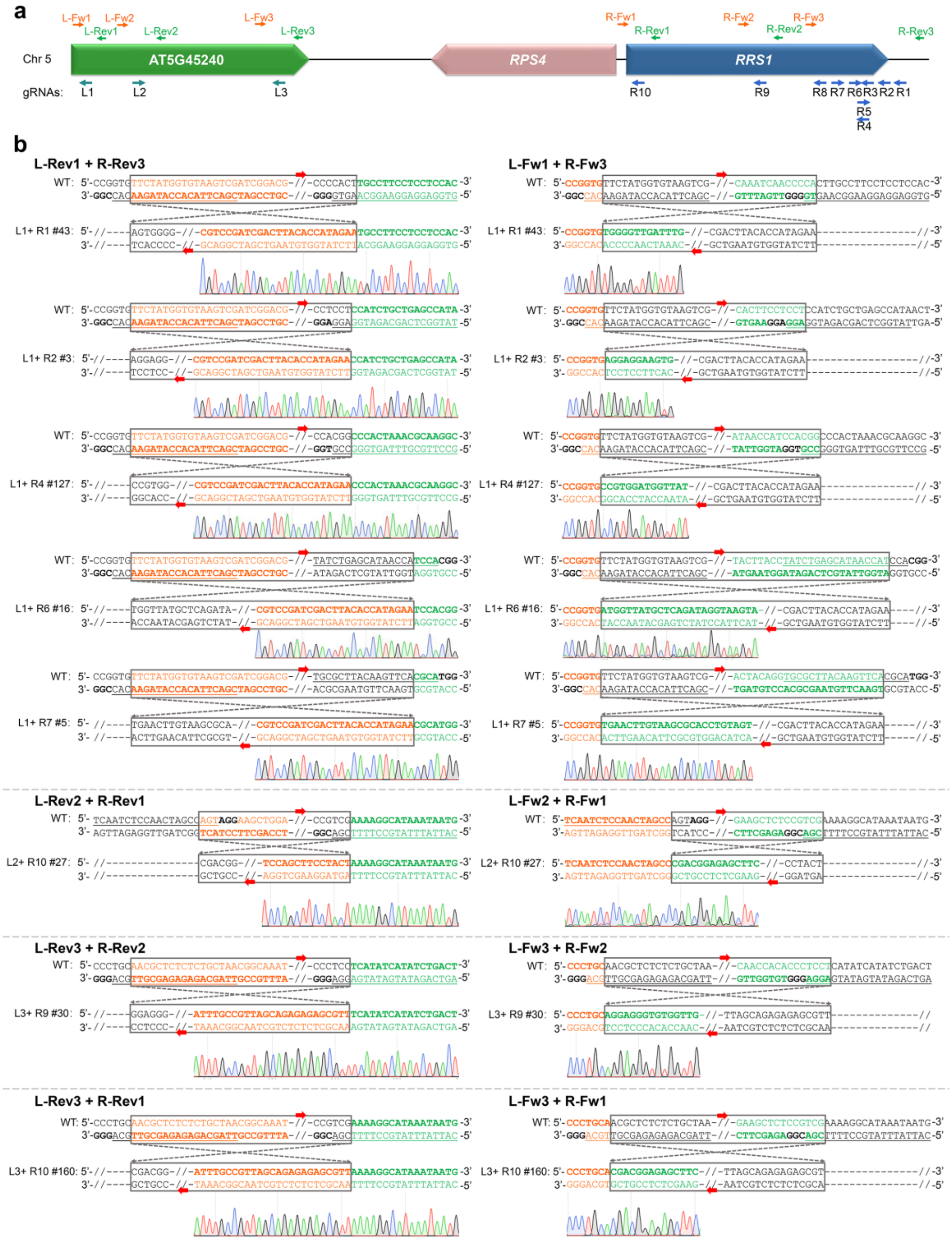

Supplementary Fig. 4. Sanger sequencing of PCR amplicons by co-aligned primers validates the inversion of AT5G45240/*AtRPS4*/*AtRRS1* mediated by different gRNA pairs.

a, Diagram of the AT5G45240/*AtRPS4*/*AtRRS1* locus with the information of gRNAs and genotyping primers. Fw, forward primer. Rev, reverse primer. **b**, Representative Sanger sequencing results of PCR amplicons using co-aligned primers validate the inversion of AT5G45240/*AtRPS4*/*AtRRS1* mediated by different gRNA-L and gRNA-R pairs. Co-aligned primers used for PCR are shown at the top.

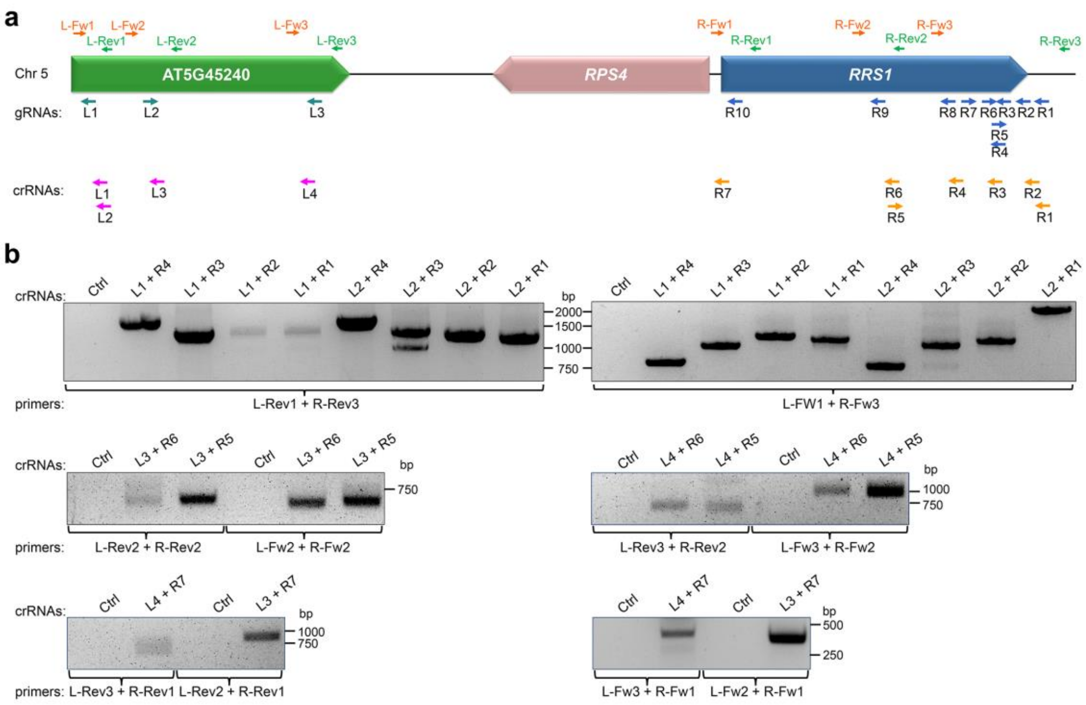

Supplementary Fig. 5. TAG inversions induced by multiplexed LbCpf1-mediated editing in *Arabidopsis* protoplasts.

a, Diagram of the AT5G45240/*AtRPS4*/*AtRRS1* locus with the information of gRNAs/crRNAs and genotyping primers. Fw, forward primer. Rev, reverse primer. Note that the crRNAs were designed to have overlapping or proximal target sites relative to gRNAs. **b**, PCR using the indicated co-aligned primers reveals that LbCpf1 in combination with different crRNA-L and crRNA-R pairs can always induce genomic inversions. LbCpf1 was transiently co-expressed with indicated crRNA-L and crRNA-R in *Arabidopsis* protoplasts. Experiments were repeated twice with similar results. Source data are provided as a Source Data file.

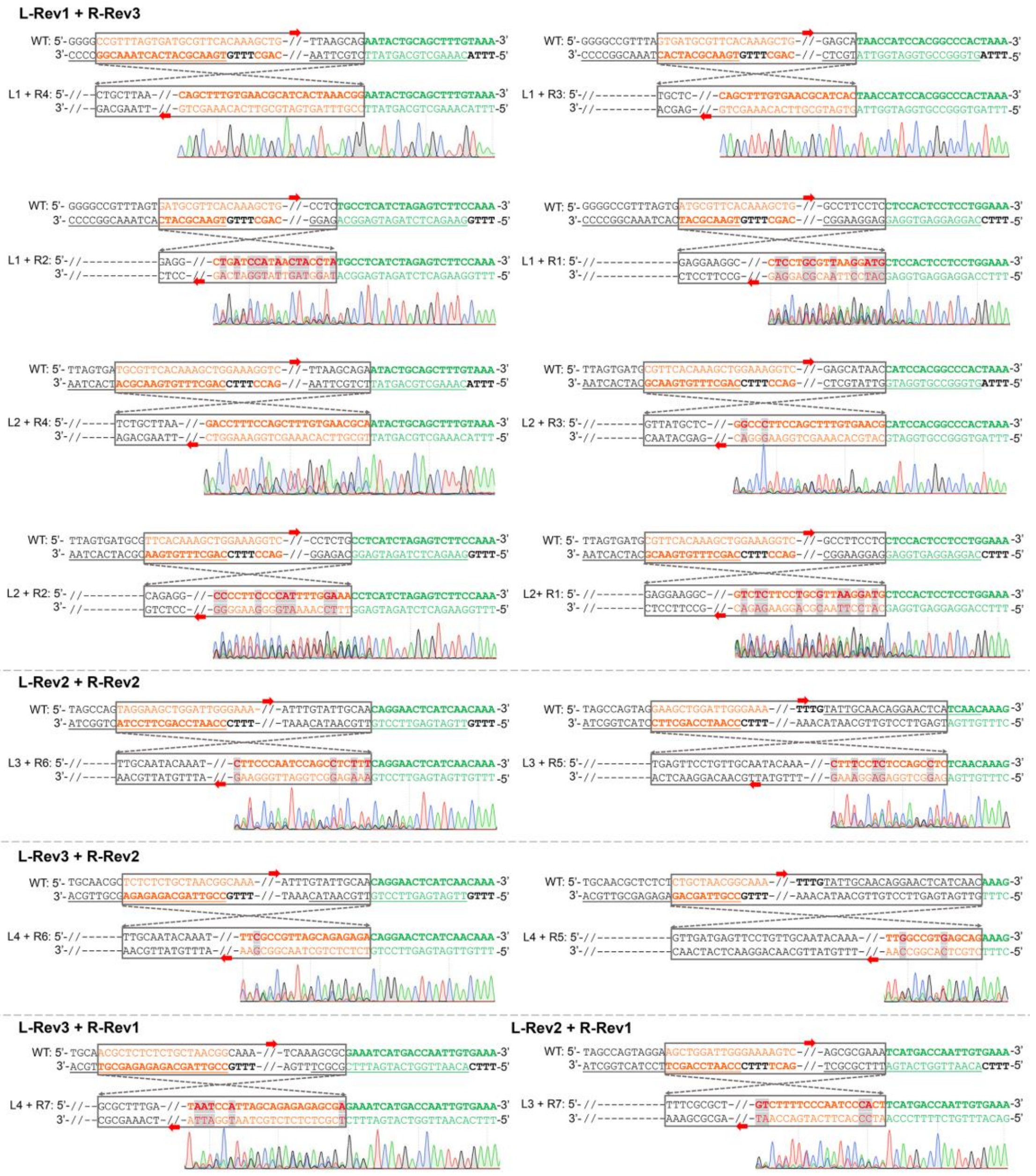

Supplementary Fig. 6. Representative Sanger sequencing results validate TAG inversions induced by multiplexed LbCpf1-mediated editing in *Arabidopsis* protoplasts.

PCR amplicons used for Sanger sequencing were shown in Supplementary Fig. 5b. Co-aligned primers used for PCR are shown at the top. Black bold letters mark PAMs. Note that heterogeneous genomic DNA from edited *Arabidopsis* protoplasts were used as PCR templates, which led to chromatographic overlapping peaks at the crRNA-induced breakpoint junction. Grey shadows highlight mismatches between expected bases and sequencing results.

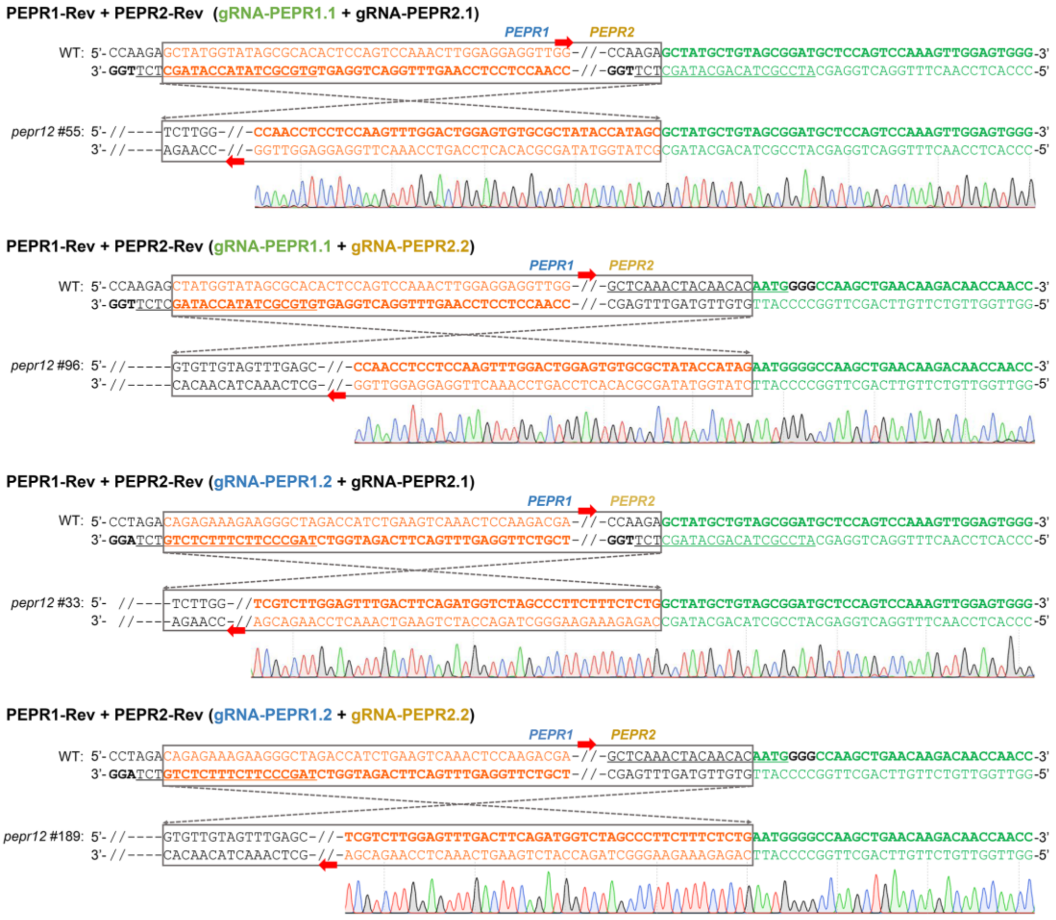

Supplementary Fig. 7. Representative Sanger sequencing results reveal four types of inversions at the *OsPEPR1*/*OsPEPR2* locus induced by *OsPEPR1*-targeting and *OsPEPR2*-targeting gRNAs in a pairwise manner.

Co-aligned primers used for PCR and gRNA pairs are shown at the top. Black bold letters mark PAMs and target sequences are underlined.

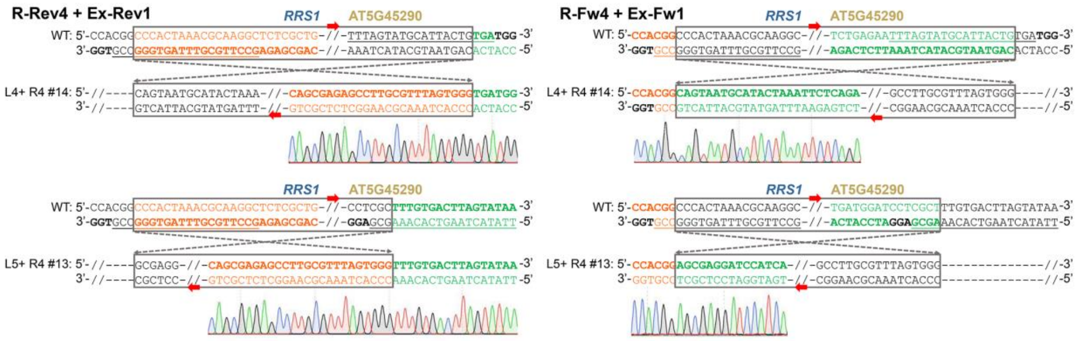

Supplementary Fig. 8. Sanger sequencing of PCR amplicons by co-aligned primers validates the inversion of *AtRRS1*/AT5G45290 mediated by different gRNA-L and gRNA-R pairs.

Co-aligned primers used for PCR are shown at the top. Black bold letters mark PAMs and target sequences are underlined.

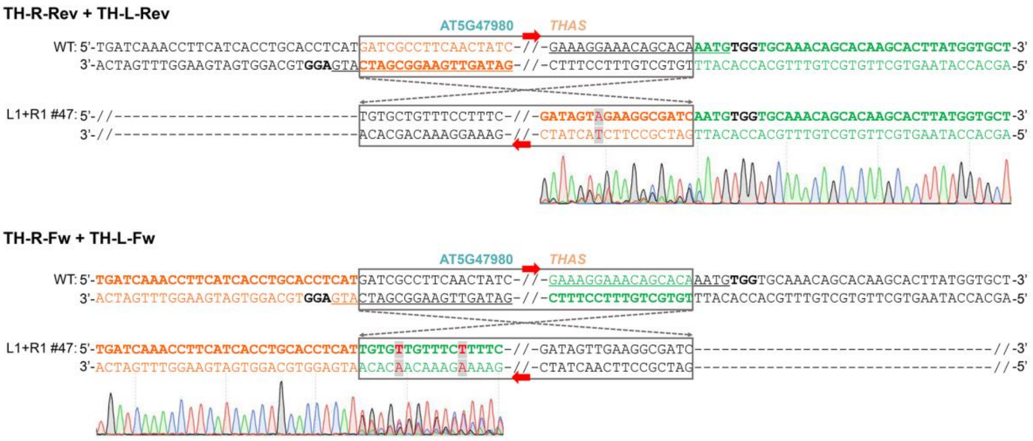

Supplementary Fig. 9. Sanger sequencing of PCR amplicons by co-aligned primers validates the inversion of triterpene biosynthetic gene cluster mediated by the gRNA-L1/gRNA-R1 pair.

Co-aligned primers used for PCR are shown at the top. Black bold letters mark PAMs and target sequences are underlined.
