# Supplementary material for: Hidden prevalence of deletion-inversion bi-alleles in CRISPR-mediated deletions of tandemly arrayed genes in plants
Source: Nat Commun. 2023 Oct 25;14:6787. doi: 10.1038/s41467-023-42490-1 (PMC10600118; doi:10.1038/s41467-023-42490-1)
Supplement: Supplementary file 5 — Reporting Summary [file 41467_2023_42490_MOESM5_ESM.pdf]

nature portfolio

Corresponding author(s): Jian-Feng Li
Last updated by author(s): Oct 8, 2023

Reporting Summary

Nature Portfolio wishes to improve the reproducibility of the work that we publish. This form provides structure for consistency and transparency in reporting. For further information on Nature Portfolio policies, see our Editorial Policies and the Editorial Policy Checklist.

Statistics

For all statistical analyses, confirm that the following items are present in the figure legend, table legend, main text, or Methods section.

n/a	Confirmed	
☐	☒	The exact sample size (*n*) for each experimental group/condition, given as a discrete number and unit of measurement
☐	☒	A statement on whether measurements were taken from distinct samples or whether the same sample was measured repeatedly
☒	☐	The statistical test(s) used AND whether they are one- or two-sided<br>*Only common tests should be described solely by name; describe more complex techniques in the Methods section.*
☒	☐	A description of all covariates tested
☒	☐	A description of any assumptions or corrections, such as tests of normality and adjustment for multiple comparisons
☐	☒	A full description of the statistical parameters including central tendency (e.g. means) or other basic estimates (e.g. regression coefficient) AND variation (e.g. standard deviation) or associated estimates of uncertainty (e.g. confidence intervals)
☒	☐	For null hypothesis testing, the test statistic (e.g. *F*, *t*, *r*) with confidence intervals, effect sizes, degrees of freedom and *P* value noted<br>*Give P values as exact values whenever suitable.*
☒	☐	For Bayesian analysis, information on the choice of priors and Markov chain Monte Carlo settings
☒	☐	For hierarchical and complex designs, identification of the appropriate level for tests and full reporting of outcomes
☐	☒	Estimates of effect sizes (e.g. Cohen's *d*, Pearson's *r*), indicating how they were calculated

Our web collection on statistics for biologists contains articles on many of the points above.

Software and code

Policy information about availability of computer code

Data collection	RNA-seq data were generated and collected on an Illumina HiSeq 4000 platform. Sanger sequencing was perform on an Applied BiosystemsTM 3730XL platform. DNA electrophoresis data were collected by the Syngene G:Box F3 gel imaging system.
Data analysis	The gRNAs were designed by using the CRISPR-GE webserver (skl.scau.edu.cn). PCR amplicons were recorded using the GeneSys image capture software (v1.3.1). Sanger sequencing data were analyzed using the SnapGene software (v7.0). The raw reads of RNA-seq were processed with a bioinformatic pipeline tool, the BMKCloud online platform (www.biocloud.net). Clean reads were mapped to the Arabidopsis TAIR10 genome assembly using the HISAT2 tool (v2.2.0). Differential expression analysis was performed using the edgeR tool (v3.6.2). Pearson correlation was calculated using the GraphPad Prism software (v6.0).

For manuscripts utilizing custom algorithms or software that are central to the research but not yet described in published literature, software must be made available to editors and reviewers. We strongly encourage code deposition in a community repository (e.g. GitHub). See the Nature Portfolio guidelines for submitting code & software for further information.

Data

Policy information about availability of data

All manuscripts must include a data availability statement. This statement should provide the following information, where applicable:

- Accession codes, unique identifiers, or web links for publicly available datasets
- A description of any restrictions on data availability
- For clinical datasets or third party data, please ensure that the statement adheres to our policy

The raw data of uncropped gels for the Figures are available in the Source Data file and those for the Supplementary Figures are provided in Supplementary Fig. 10. The CRISPR target sequences and PCR primer sequences are included in the Supplementary Data files. The RNA-seq data generated in this study have been deposited in the Sequence Read Archive (SRA) database under accession code PRJNA923581 [https://www.ncbi.nlm.nih.gov/sra/?term=PRJNA923581]. Source data are provided with this paper.

Research involving human participants, their data, or biological material

Policy information about studies with human participants or human data. See also policy information about sex, gender (identity/presentation), and sexual orientation and race, ethnicity and racism.

Reporting on sex and gender	Not applicable.
Reporting on race, ethnicity, or other socially relevant groupings	Not applicable.
Population characteristics	Not applicable.
Recruitment	Not applicable.
Ethics oversight	Not applicable.

Note that full information on the approval of the study protocol must also be provided in the manuscript.

Field-specific reporting

Please select the one below that is the best fit for your research. If you are not sure, read the appropriate sections before making your selection.

- [x] Life sciences
- [ ] Behavioural & social sciences
- [ ] Ecological, evolutionary & environmental sciences

For a reference copy of the document with all sections, see nature.com/documents/nr-reporting-summary-flat.pdf

Life sciences study design

All studies must disclose on these points even when the disclosure is negative.

Sample size	No sample size calculation was performed to predetermine sample size. Two biological repeats showing similar results generally allowed a conclusion to be made on PCR-based genotyping experiments. For experiments with increased variations or requiring in-depth evaluation, such as evaluating the delinver mutation frequencies among transgenic plants with different TAG loci being targeted, we assayed all the transgenic Arabidopsis plants or transgenic rice calli we could obtain. Based on generally accepted plant sample sizes (i.e., dozens to hundreds) in relevant publications in this field, a total of ~2,650 transgenic events assayed in this study were more than sufficient for us to conclude that the delinver mutations occur frequently in plants.
Data exclusions	No data were excluded from the analyses.
Replication	For RNA-seq analysis, two biological replicates using distinct samples were performed. All attempts at replication were successful.
Randomization	Samples were randomly allocated into experimental groups.
Blinding	The investigators were completely blinded to group allocation during data collection and/or analysis.

Reporting for specific materials, systems and methods

We require information from authors about some types of materials, experimental systems and methods used in many studies. Here, indicate whether each material, system or method listed is relevant to your study. If you are not sure if a list item applies to your research, read the appropriate section before selecting a response.

Materials & experimental systems

n/a	Involved in the study
☒	☐ Antibodies
☒	☐ Eukaryotic cell lines
☒	☐ Palaeontology and archaeology
☒	☐ Animals and other organisms
☒	☐ Clinical data
☒	☐ Dual use research of concern
☐	☒ Plants

Methods

n/a	Involved in the study
☒	☐ ChIP-seq
☒	☐ Flow cytometry
☒	☐ MRI-based neuroimaging

Dual use research of concern

Policy information about dual use research of concern

Hazards

Could the accidental, deliberate or reckless misuse of agents or technologies generated in the work, or the application of information presented in the manuscript, pose a threat to:

No	Yes
☒	☐ Public health
☒	☐ National security
☒	☐ Crops and/or livestock
☒	☐ Ecosystems
☒	☐ Any other significant area

Experiments of concern

Does the work involve any of these experiments of concern:

No	Yes
☒	☐ Demonstrate how to render a vaccine ineffective
☒	☐ Confer resistance to therapeutically useful antibiotics or antiviral agents
☒	☐ Enhance the virulence of a pathogen or render a nonpathogen virulent
☒	☐ Increase transmissibility of a pathogen
☒	☐ Alter the host range of a pathogen
☒	☐ Enable evasion of diagnostic/detection modalities
☒	☐ Enable the weaponization of a biological agent or toxin
☒	☐ Any other potentially harmful combination of experiments and agents
