# Supplementary material for: Hidden prevalence of deletion-inversion bi-alleles in CRISPR-mediated deletions of tandemly arrayed genes in plants
Source: Nat Commun. 2023 Oct 25;14:6787. doi: 10.1038/s41467-023-42490-1 (PMC10600118; doi:10.1038/s41467-023-42490-1)

Uncropped gels for Figure 2c

Fig 2c 1st panel

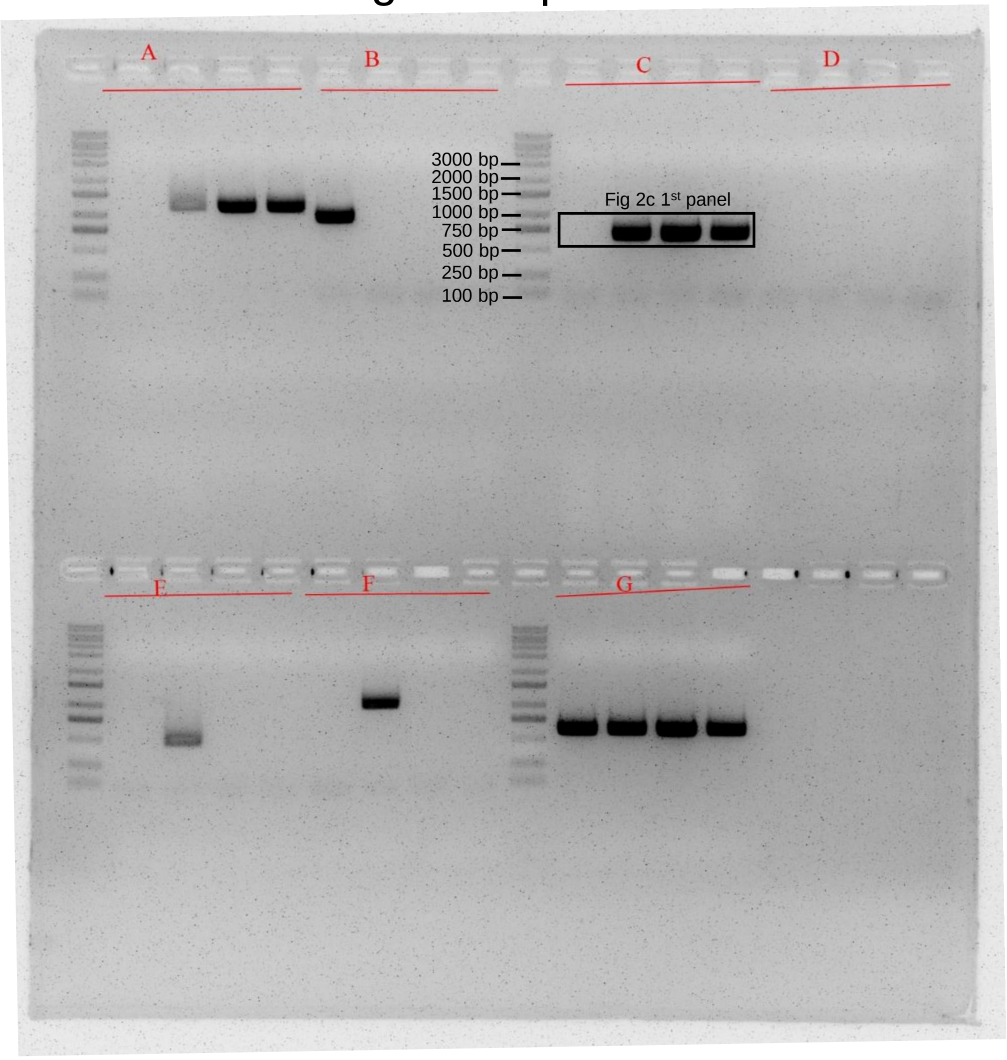

Uncropped gels for Figure 2c

Fig 2c 2nd-4th panels

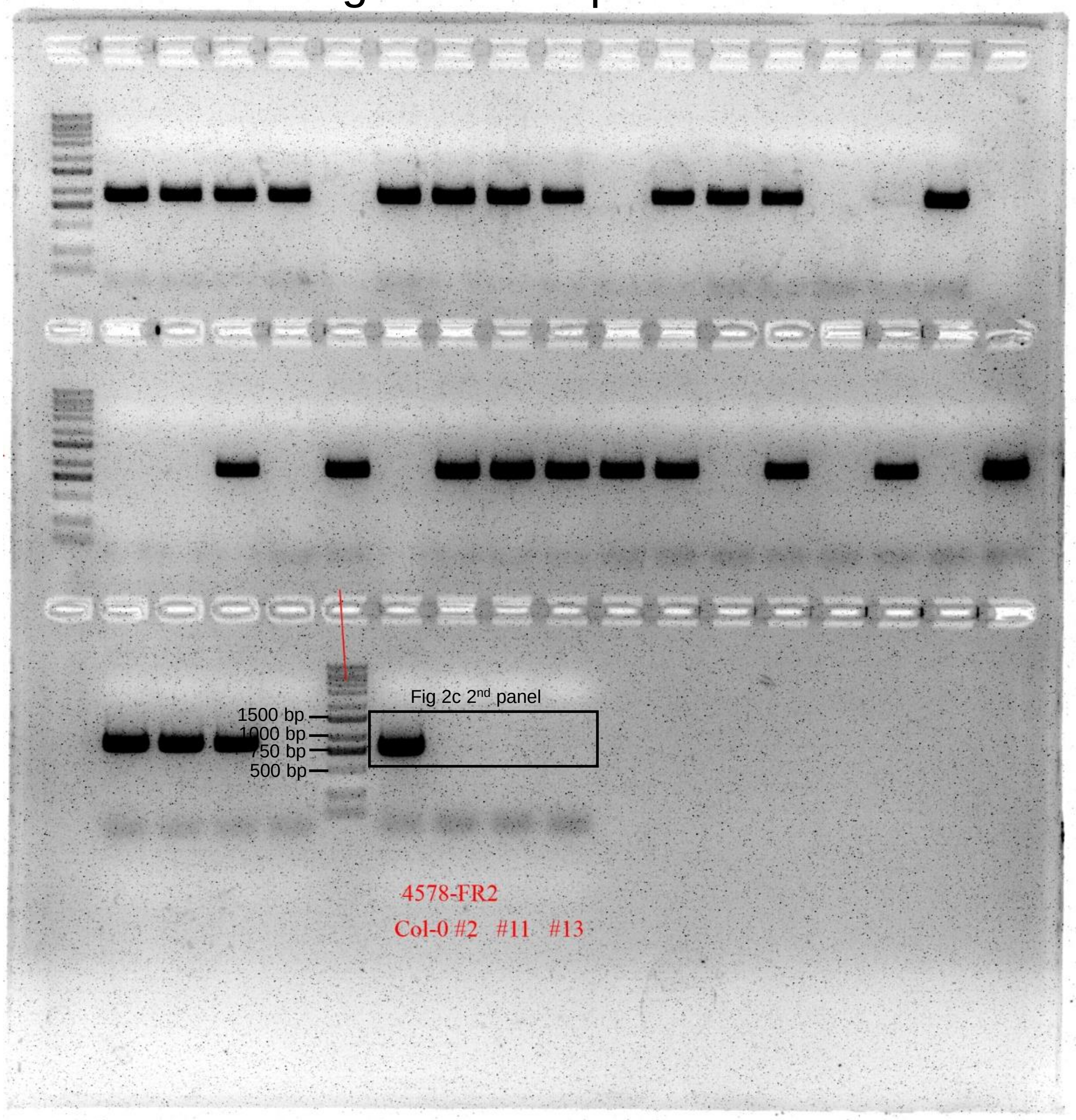

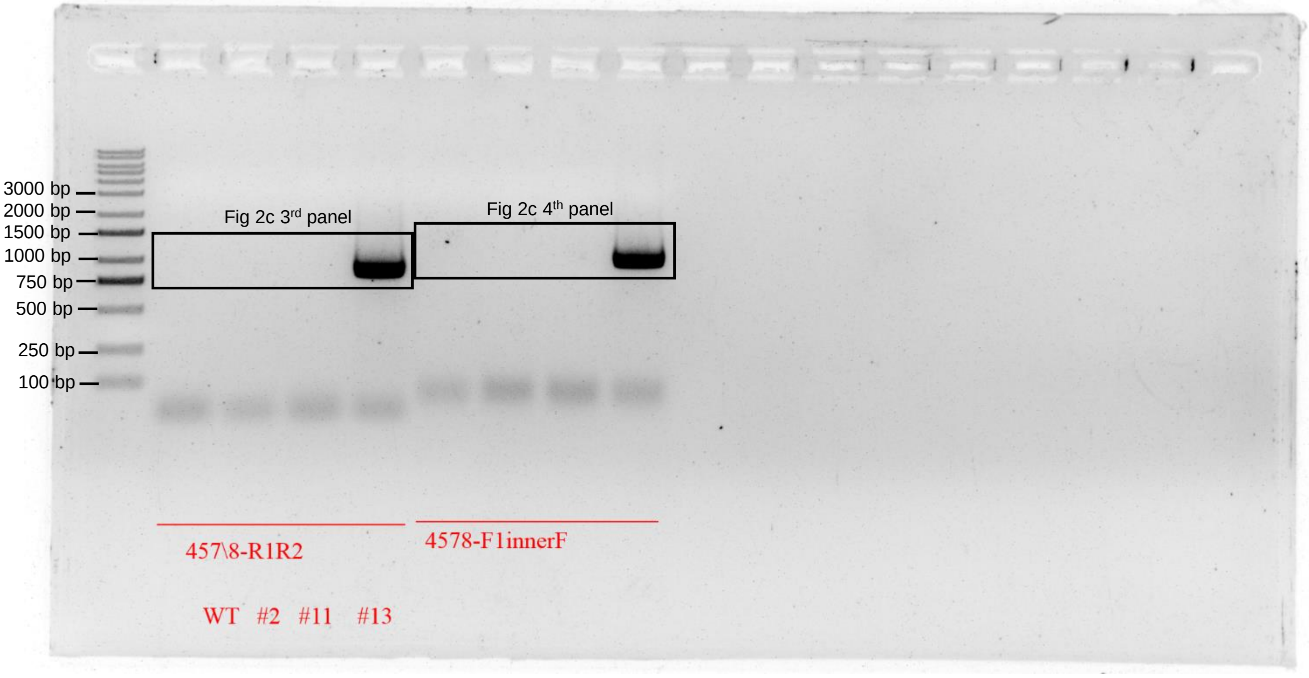

Supplement: Supplementary file 6 — Source Data [file 41467_2023_42490_MOESM6_ESM.zip › Uncropped gels for Figure 2c.pdf]
