# Supplementary figures and images for: Hidden prevalence of deletion-inversion bi-alleles in CRISPR-mediated deletions of tandemly arrayed genes in plants
Source: Nat Commun. 2023 Oct 25;14:6787. doi: 10.1038/s41467-023-42490-1 (PMC10600118; doi:10.1038/s41467-023-42490-1)

Uncropped gels for Figure 1f

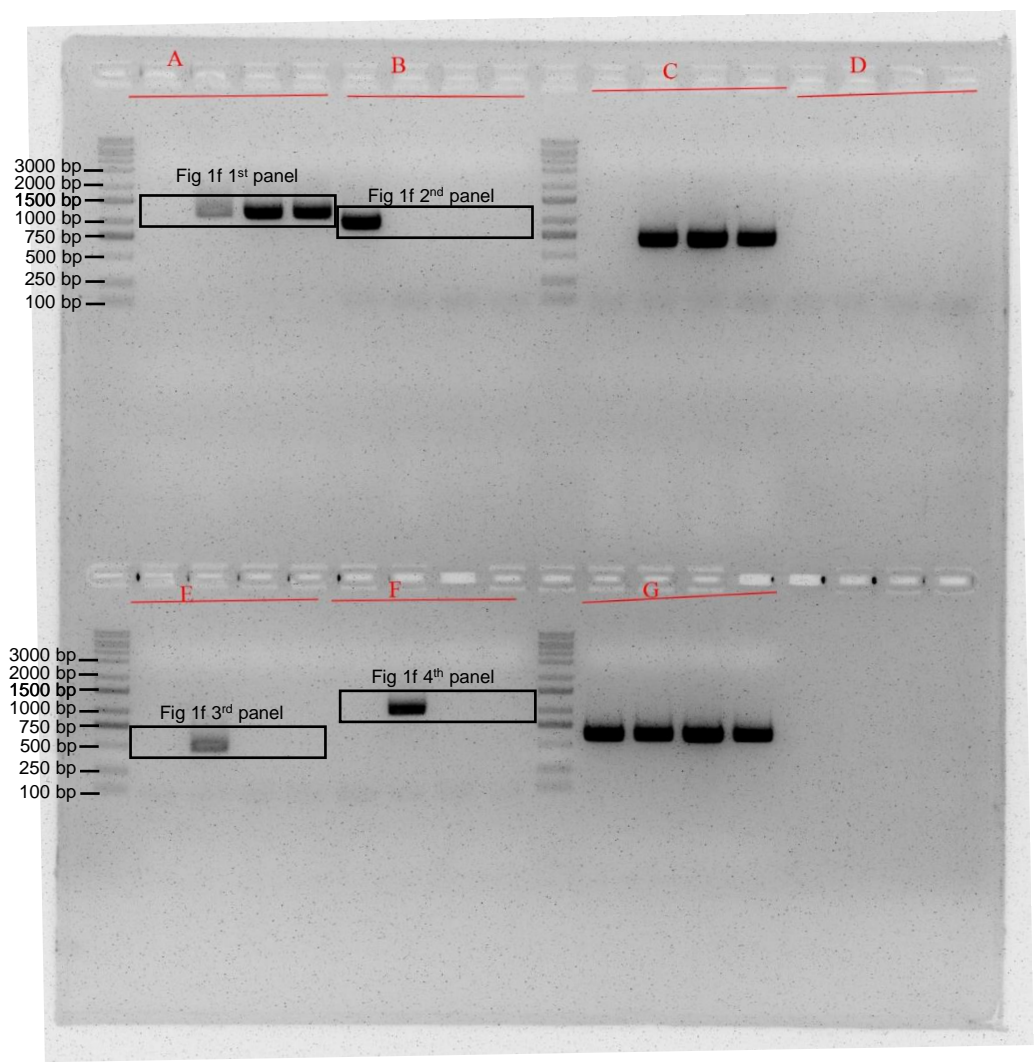

Supplement: Supplementary file 6 — Source Data [file 41467_2023_42490_MOESM6_ESM.zip › Uncropped gels for Figure 1f.pdf]

# Uncropped gels for Figure 3a

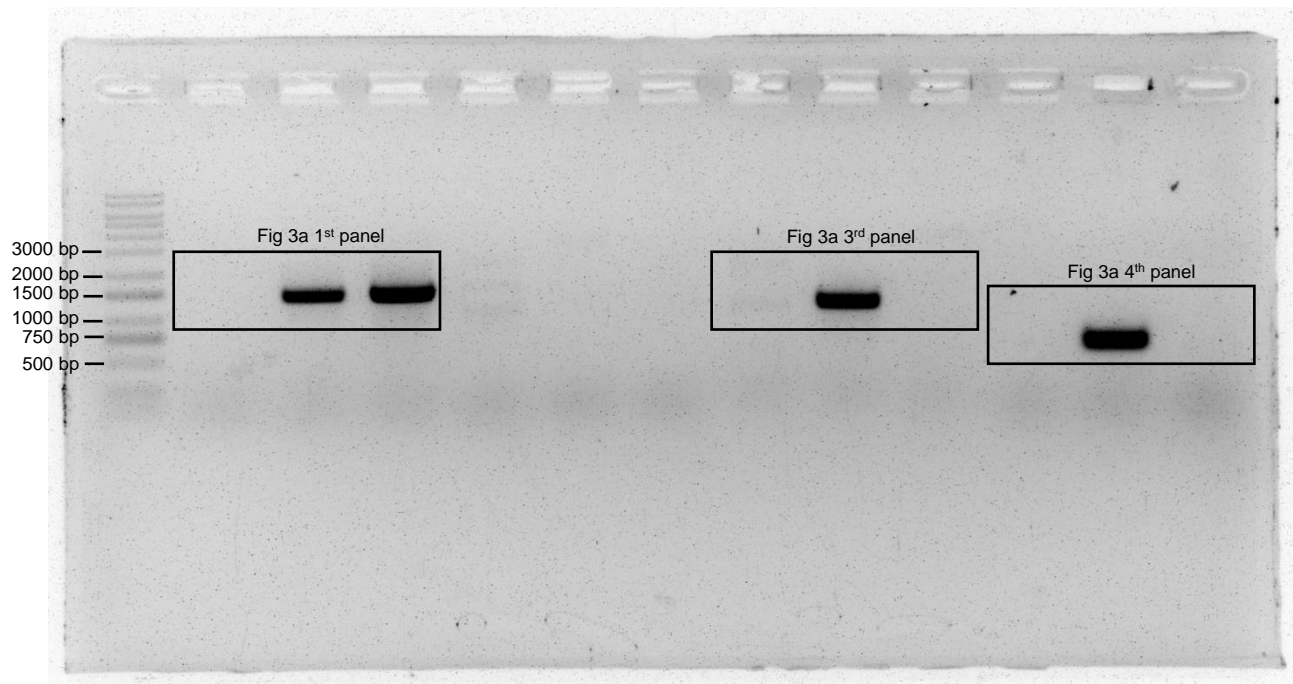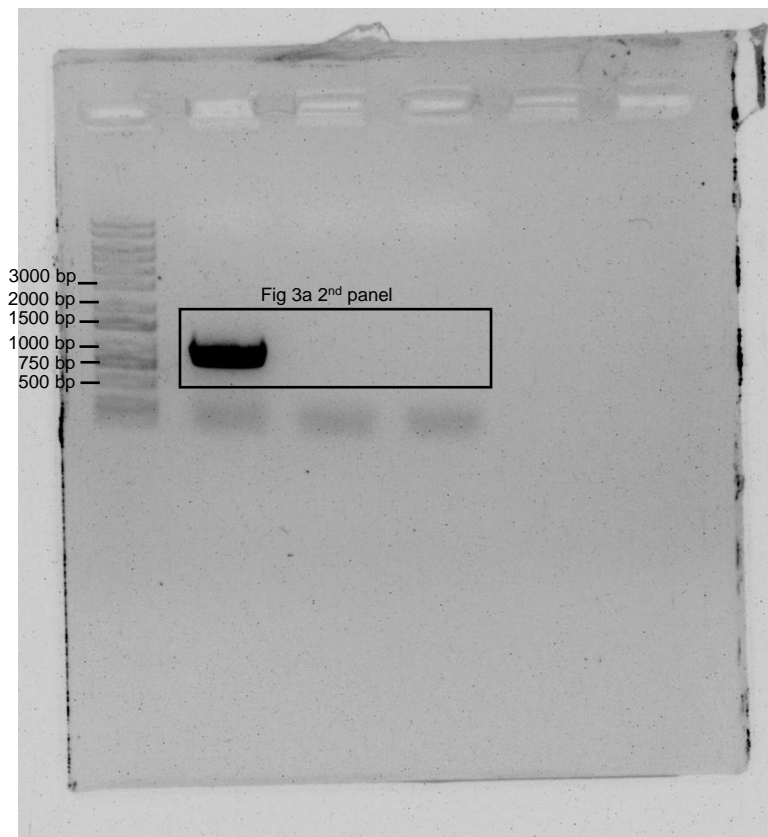

Supplement: Supplementary file 6 — Source Data [file 41467_2023_42490_MOESM6_ESM.zip › Uncropped gels for Figure 3a.pdf]

Uncropped gels for Figure 3b

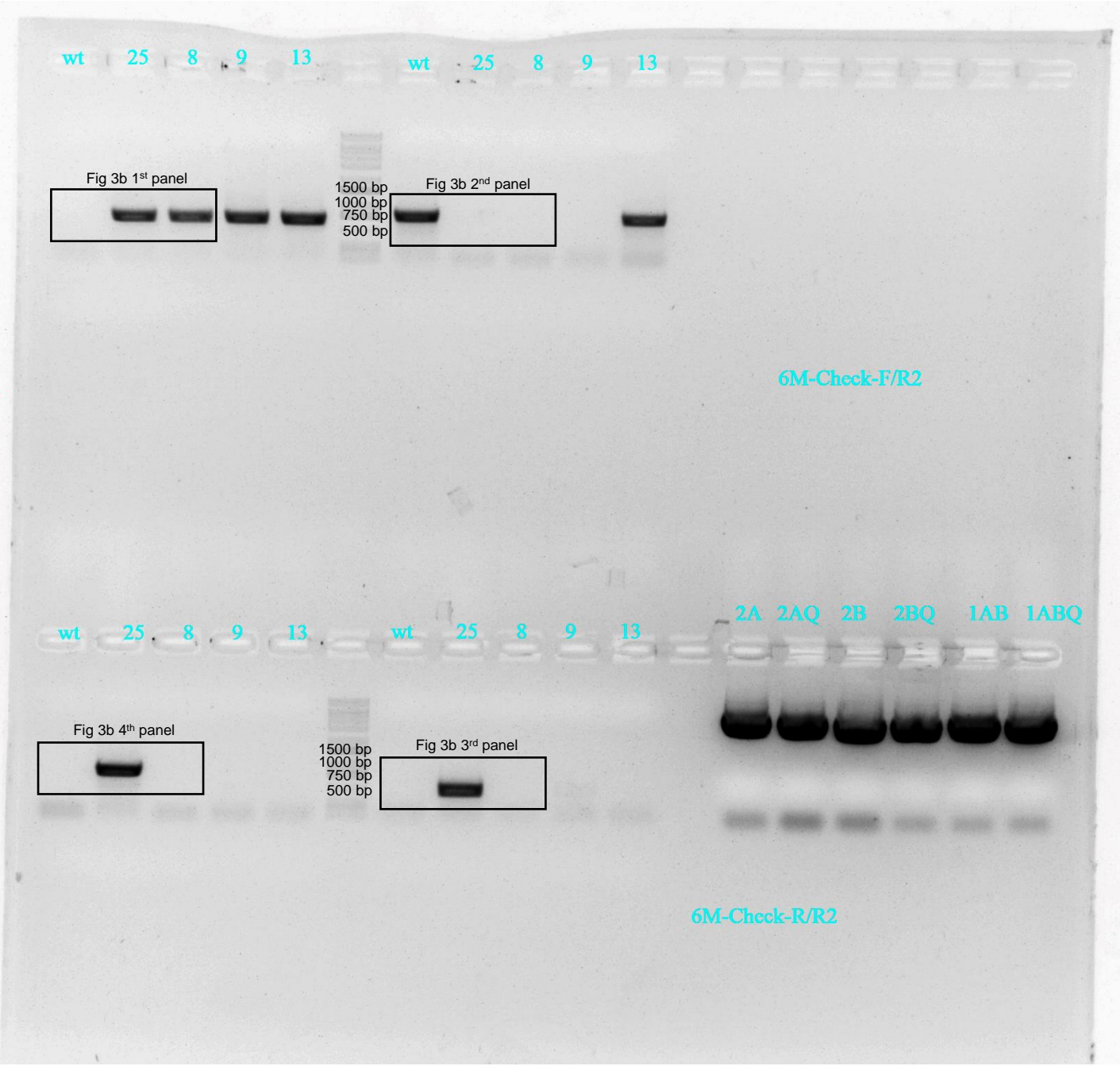

Supplement: Supplementary file 6 — Source Data [file 41467_2023_42490_MOESM6_ESM.zip › Uncropped gels for Figure 3b.pdf]

Uncropped gels for Supplementary Figure 1a

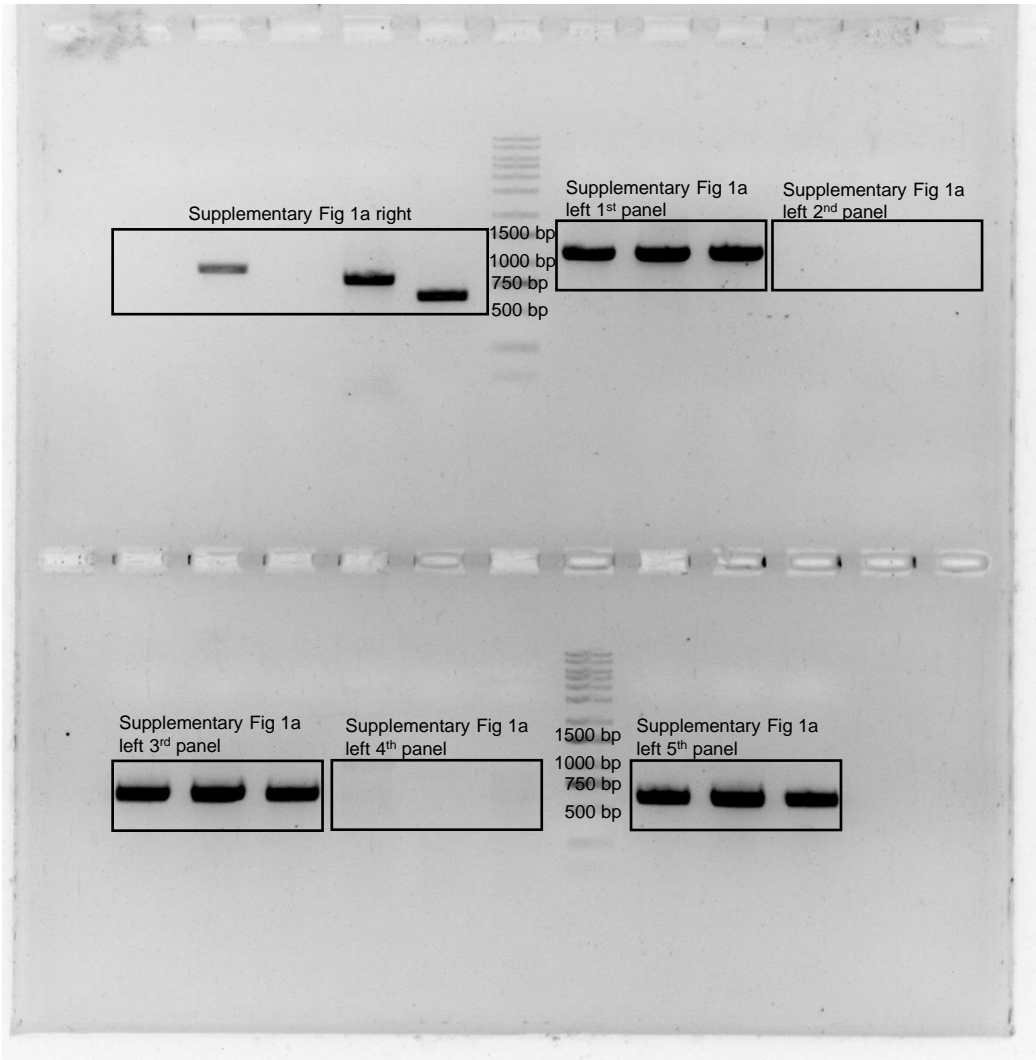

Supplement: Supplementary file 6 — Source Data [file 41467_2023_42490_MOESM6_ESM.zip › Uncropped gels for Supplementary Figure 1a.pdf]

Uncropped gels for Supplementary Figure 2a

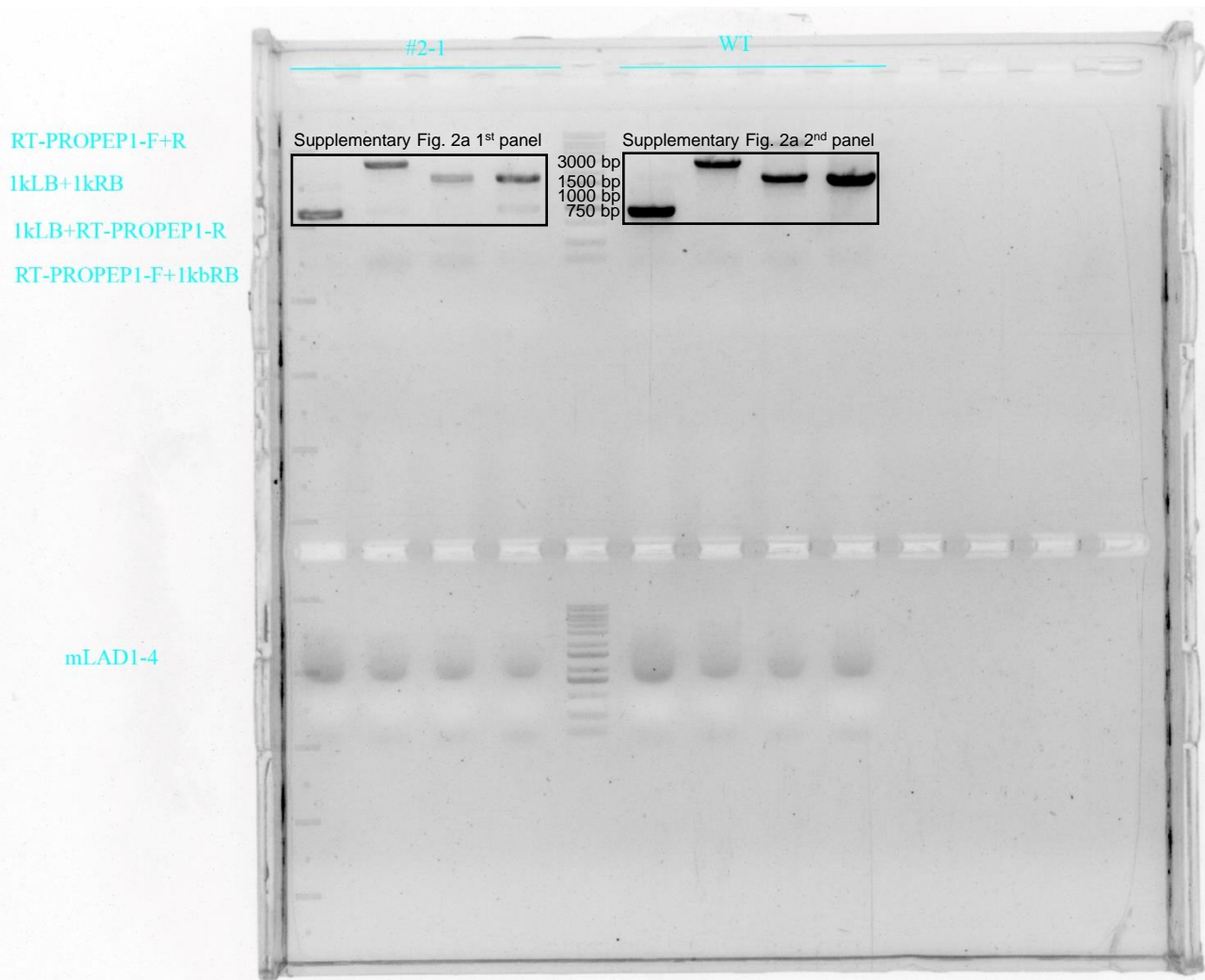

Supplement: Supplementary file 6 — Source Data [file 41467_2023_42490_MOESM6_ESM.zip › Uncropped gels for Supplementary Figure 2a.pdf]

Supplementary Fig. 2c left

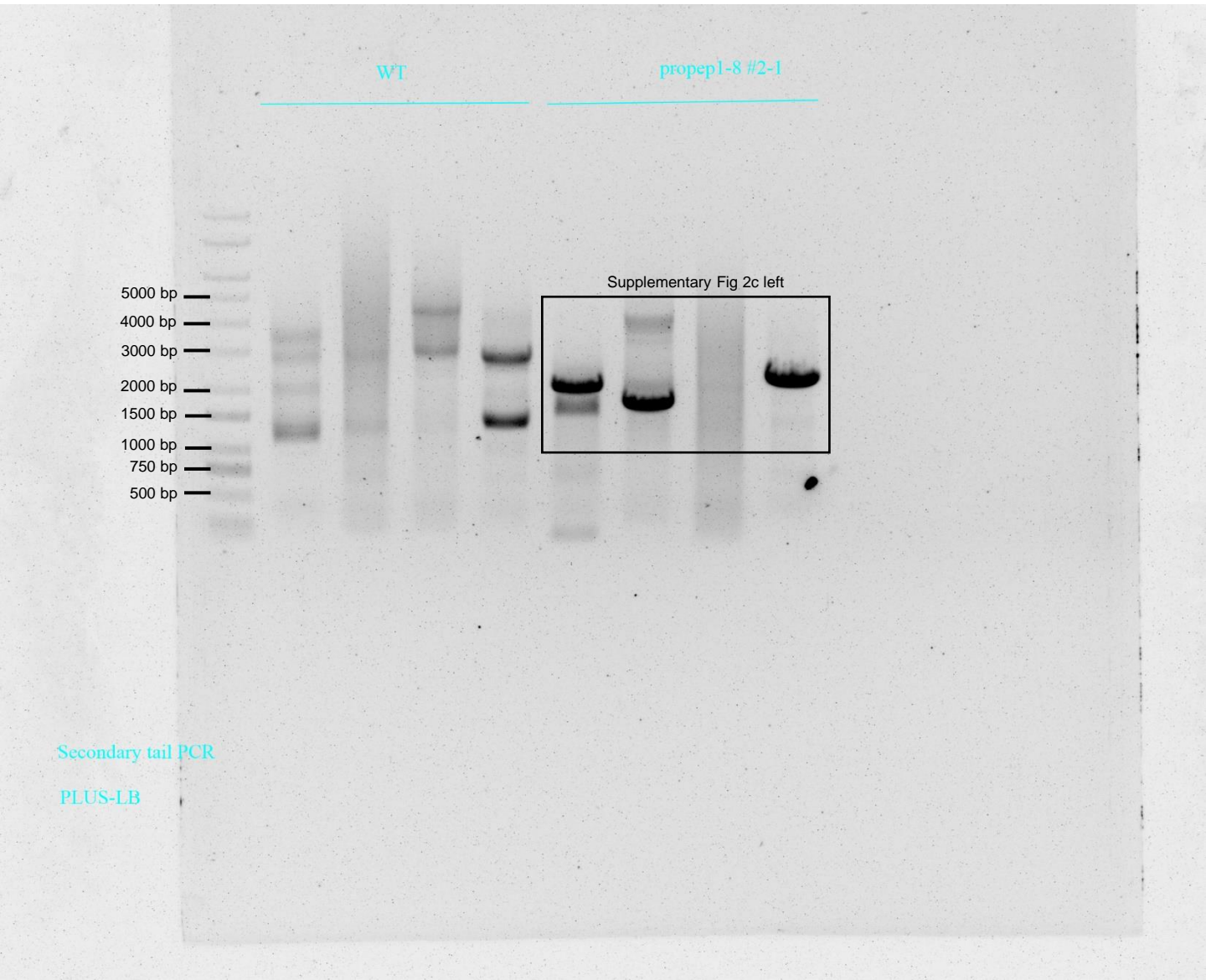

Supplementary Fig. 2c right

Secondary

Primary

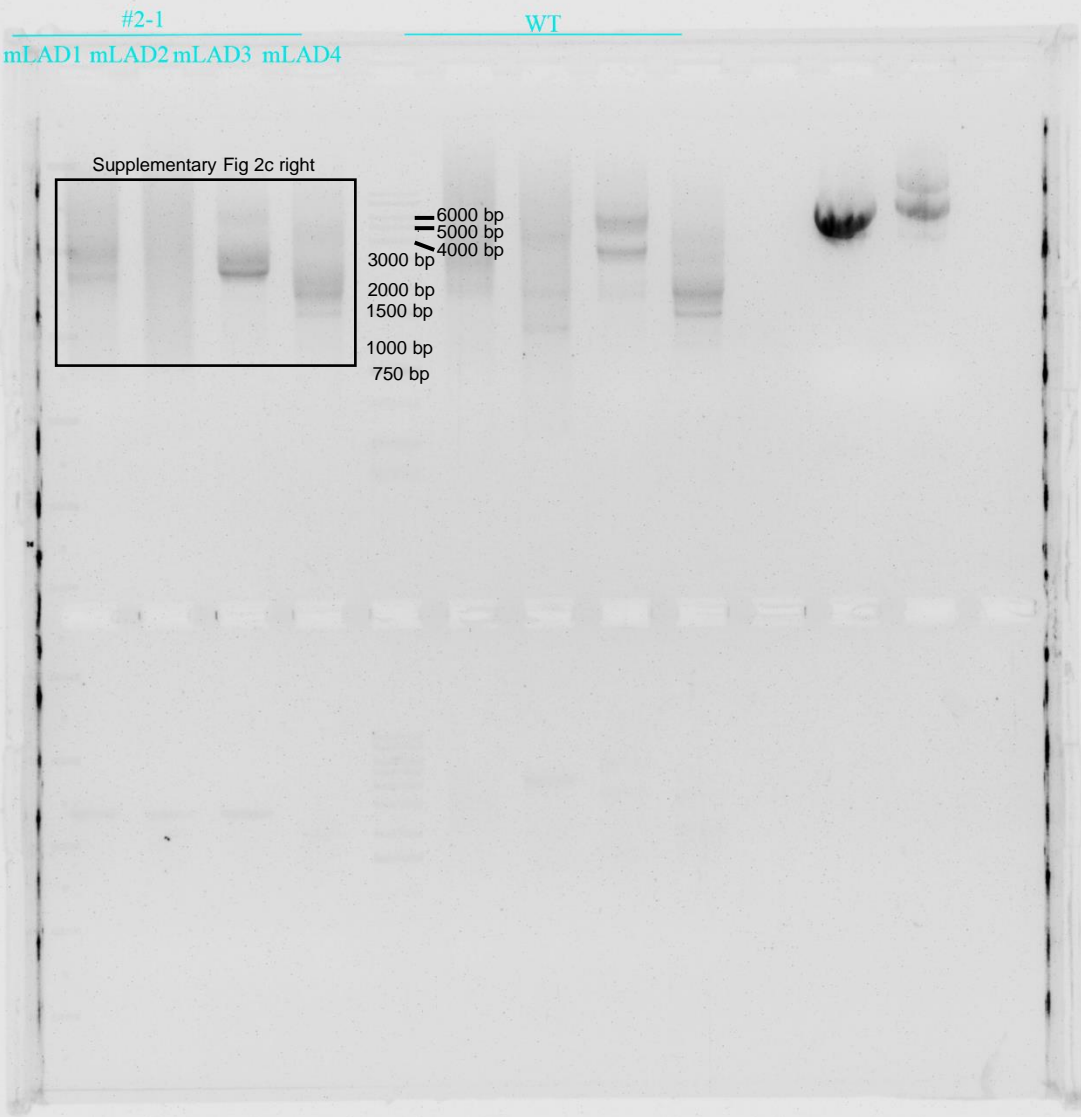

Supplement: Supplementary file 6 — Source Data [file 41467_2023_42490_MOESM6_ESM.zip › Uncropped gels for Supplementary Figure 2c.pdf]
